# Drug-targeted endoscopic submucosal dissection: a hypothetical framework toward non-electromechanical mucosal resection

**DOI:** 10.3389/fmed.2025.1668295

**Published:** 2025-11-25

**Authors:** Conghua Song, Huiyuan Xu, Xiaomei Li

**Affiliations:** 1 Gastrointestinal Endoscopy Center, The Affiliated Hospital of Putian University, Putian, Fujian, China; 2 School of Basic Medicine, Putian University, Putian, Fujian, China; 3 Key Laboratory of Translational Tumor Medicine in Fujian Province, Putian University, Putian, Fujian, China

**Keywords:** endoscopic mucosal resection (EMR), endoscopic submucosal dissection (ESD), submucosal injection, superficial gastrointestinal neoplasms, therapeutic endoscopy

## Abstract

Endoscopic submucosal dissection (ESD) is a widely used technique for *en bloc* resection of gastrointestinal neoplasms. However, it is technically demanding, time-consuming, and associated with risks such as perforation and bleeding, primarily due to the reliance on electromechanical dissection. In this theoretical proposal, we introduce a novel concept termed “drug-targeted endoscopic submucosal dissection (DT-ESD),” which aims to partially replace mechanical dissection by localized chemical degradation of the submucosa. The proposed method preserves most procedural steps of conventional ESD but incorporates selective enzymatic digestion to facilitate submucosal separation. This approach has the potential to reduce procedure time, thermal injury, and technical complexity. We detail the conceptual workflow, candidate agents, delivery strategy, potential advantages, and risks. Though theoretical at this stage, DT-ESD represents a potential paradigm shift in endoscopic therapy. Further validation *in ex vivo* and *in vivo* models is needed. If validated, DT-ESD could transform the paradigm of minimally invasive gastrointestinal therapy and could represent a transformative advancement in therapeutic endoscopy.

## 1 Introduction

Endoscopic submucosal dissection (ESD) has transformed the treatment of superficial gastrointestinal neoplasms by enabling *en bloc* resection with histologically negative margins. However, its clinical application is limited by a steep learning curve, lengthy procedural time, and significant risks of perforation, bleeding, and thermal injury. Recent studies estimate that the learning curve for competent ESD performance requires over 30–50 supervised cases, with procedure times often exceeding 90–120 min for large lesions, and complication rates including perforation (4%–10%) and delayed bleeding (up to 15%), particularly in fibrotic or anatomically difficult locations (1–3). These drawbacks primarily result from the technical challenge of dissecting the submucosal layer using electrosurgical instruments.

Anatomically, the gastrointestinal wall comprises four layers: mucosa, submucosa, muscularis propria, and serosa. The submucosa is structurally distinct, characterized by a dense extracellular matrix (ECM) rich in type I and III collagen, hyaluronic acid, fibroblasts, vasculature, and nerve plexuses. This ECM-rich composition renders it particularly susceptible to enzymatic degradation, unlike the overlying mucosa and underlying muscularis propria, which possess distinct cellular and matrix architecture. These histological differences provide a rationale for developing pharmacological agents that selectively target the submucosa.

Building on this concept, we propose a theoretical technique—drug-targeted endoscopic submucosal dissection (DT-ESD)—that employs localized enzyme injection to loosen the submucosal layer and facilitate dissection. The feasibility of this approach is supported by the clinical use of collagenase clostridium histolyticum (Xiaflex^®^), which has demonstrated high efficacy and safety in the enzymatic treatment of Dupuytren’s contracture and Peyronie’s disease. In phase III trials, Xiaflex^®^ achieved significant contracture release in over 60% of patients with minimal systemic side effects, primarily through localized collagen degradation within fibrous plaques, supporting its potential application in endoluminal ECM remodeling (4, 5). The established clinical efficacy and safety of localized collagenase injection (e.g., Xiaflex^®^) for fibrotic conditions like Dupuytren’s contracture provides a direct translational precedent, demonstrating that controlled enzymatic digestion of the extracellular matrix is a viable strategy in human soft tissue and can be repurposed for submucosal dissection within the gastrointestinal tract.

## 2 About hypothesis and theory of DT-ESD

### 2.1 Theoretical concept and workflow

**Figure 1:**
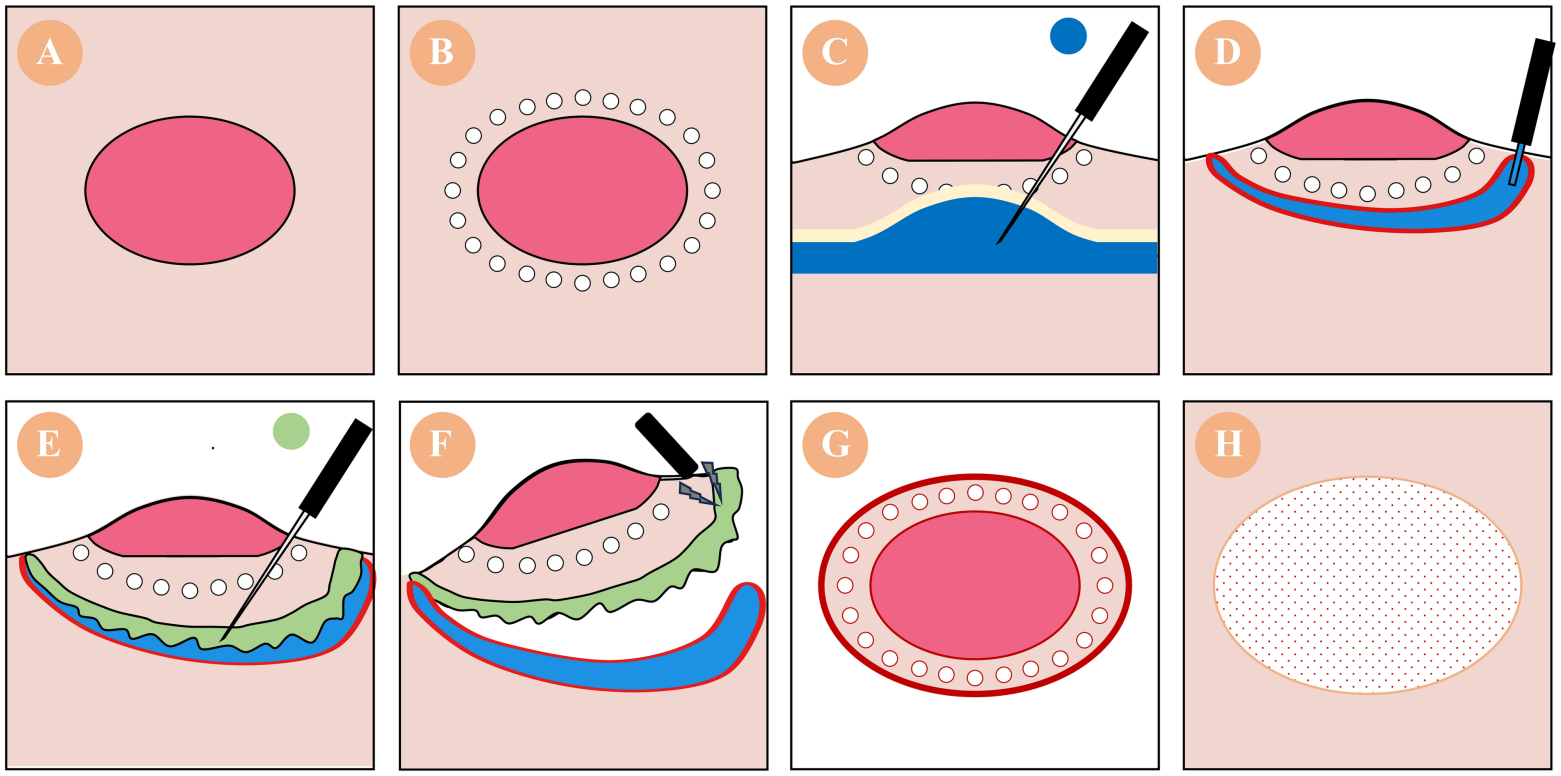
Schematic workflow of drug-targeted endoscopic submucosal dissection (DT-ESD). **(A)** Lesion identification. **(B)** Circumferential boundary incision. **(C)** Submucosal elevation via saline injection to create a lifting cushion (blue). **(D)** Localized enzymatic injection into the submucosa. **(E)** Enzymatic digestion of submucosal ECM facilitates tissue loosening (green). **(F)** Completion of dissection using blunt or minimal electrosurgical separation. **(G)** Intact *en bloc* specimen following resection. **(H)** Post-resection ulcer bed showing preserved deep layers. DT-ESD is currently a theoretical construct. Clinical performance projections are based on anticipated advantages of enzyme-assisted tissue separation.

The DT-ESD technique maintains the core structure of ESD but incorporates one key innovation: the selective injection of a submucosa-dissolving agent after initial mechanical isolation. As illustrated in Figure 1, the DT-ESD workflow comprises six main sequential steps:

- Step 1: Mucosal marking
  The lesion is marked with dye, image enhanced endoscopy (IEE), or a coagulation technique to delineate the boundaries.
- Step 2: Submucosal injection
  We propose a refinedtwo-step injection protocol inspired by endoscopic carbon-nanoparticle tattooing. First, inject normal saline—optionally tinted with indigo carmine—to create a robust submucosal cushion. This pre-lift achieves two goals: it hydrodissects and expands the submucosal space while elevating the lesion, thereby separating the mucosa from the muscularis propria and establishing a well-defined, safer compartment for the next step. Second, with this compartment in place, the enzyme formulation can be delivered in a controlled manner into the submucosa; the pre-lift limits downward diffusion toward the muscularis propria and thus reduces the risk of full-thickness injury.
- Step 3: Boundary formation (mucosal incision)
  A circumferential incision is made around the lesion using a standard endoscopic knife. Importantly, both the mucosa and superficial submucosa are incised to create a relatively closed boundary, which serves to confine the subsequent action of the enzymatic agent and prevent undesired spread to adjacent normal tissue.
- Step 4: Targeted enzymatic injection (enzyme-assisted dissection)
  With a pre-expanded, fluid-filled submucosal cushion established, the enzyme formulation is delivered into this confined compartment under direct endoscopic visualization. The cushion acts as a mechanical buffer against inadvertent deep needle placement and helps restrict early vertical diffusion, thereby lowering the risk of intramuscular injection.

A biologically active agent, such as collagenase, hyaluronidase, or a combination thereof, is injected into the pre-lifted submucosal space (Step 2). The aim is to enzymatically degrade extracellular matrix components (collagen, elastin, hyaluronic acid) within the submucosa, thereby achieving tissue loosening or partial liquefaction.

The injection is delivered through a standard 19-23G endoscopic injection needle, with an estimated volume of 2–5 mL per lesion quadrant, adjusted by lesion size and location. Injection duration per site is approximately 5–10 s to ensure localized distribution.

Without removing the endoscopically positioned injection needle, the syringe containing the saline is disconnected and replaced with a syringe containing the pre-prepared enzyme formulation. The enzyme is then injected into the pre-established submucosal fluid cushion. This seamless transition ensures that the enzymatic agent is delivered precisely into the intended dissection plane, maximizing localization and safety. The subsequent injection follows the “multi-point, low-volume” strategy. Instead of a single, large-volume bolus, the enzyme formulation will be delivered through numerous, small aliquots (e.g., 0.5–1.0 mL per injection point) distributed across the submucosal plane beneath the lesion. This technique, standard in other endoscopic therapies, promotes more even dispersion.

To maximize safety and confine the enzymatic activity to the submucosa, the solution should be injected slowly into the elevated layer under direct endoscopic visualization, preferably with co-injected dye (e.g., indigo carmine) to monitor spread.

The selected enzymes may be formulated in a viscous vehicle (e.g., hydrogel-based buffer) to reduce vertical diffusion. Following a short incubation period (typically an initial estimate of 3–5 min, titrated to the visual endpoint of tissue loosening), enzymatic disruption facilitates subsequent dissection. The optimal duration is agent-specific and will be determined through preclinical studies.

The proposed 3–5 min window is an initial estimate, and we envision a more sophisticated, dynamic approach in practice:

- A titratable and visual endpoint: Rather than a rigid, fixed time, the optimal incubation period will be lesion-specific and guided by real-time visual feedback. The endpoint will be the observed “submucosal lift sign”—the moment under endoscopic view when the lesion begins to separate freely or a loss of resistance is noted with blunt probing. This is analogous to the surgical concept of “dissecting the plane,” where the procedure follows anatomical changes rather than a timer.
- Dependence on formulation kinetics: The required incubation time is intrinsically linked to the specific activity of the final enzyme formulation and its vehicle. A primary goal of our proposed preclinical screening pipeline is to define the dose–response and time-action profile of candidate agents. This will allow us to select or engineer enzymes with the most favorable kinetic profile (rapid onset, self-limiting action). A key aim of the preclinical pipeline is to define dose–response and time-action profiles, enabling selection or engineering of formulations with rapid onset and self-limiting kinetics.
- Integration with the safety strategy: It is crucial to reiterate that the incubation time is not the sole determinant of safety. The rapid-deactivation protocol is designed precisely to terminate enzymatic activity at the end of the chosen incubation period, effectively decoupling the dissection time from the risk of prolonged, uncontrolled enzyme action. This allows for a more flexible and effective incubation window. The rapid-deactivation protocol is a critical companion to this timing. It ensures that once the desired tissue loosening is achieved (whether at 3 or 8 min), the enzymatic process can be actively and immediately terminated, thereby mitigating the risks associated with prolonged exposure.

- Step 5: Remove the lesion
  Once the submucosa has been sufficiently disrupted, the lesion can be more easily separated and removed with foreign body forceps or endoloop, using either minimal electrosurgical assistance or blunt traction techniques when necessary.

### 2.2 Candidate agents for submucosal digestion

To achieve effective and selective submucosal dissection, pharmacological agents must be capable of degrading key components of the extracellular matrix (ECM) within the submucosal layer, particularly collagen and glycosaminoglycans. The selection of such agents should be based on their substrate specificity, enzymatic potency, and compatibility with endoscopic delivery systems. Several enzymes, either used individually or in combination, have been identified as potential candidates due to their ability to disrupt ECM architecture. In addition, drug delivery may be enhanced through use of hydrogel vehicles, nanoparticles, or controlled-release microspheres, minimizing diffusion to adjacent layers. A summary of these candidate agents and their corresponding target matrices is provided in Table 1.

**Table 1:** Candidate agents for submucosa-specific degradation.

Enzyme	Target matrix	Application rationale
Collagenase	Type I and III collagen	Directly breaks down the major ECM framework of submucosa
Hyaluronidase	Hyaluronic acid	Facilitates matrix loosening and fluid spread
Elastase	Elastin	May assist in deeper tissue separation in select sites
Combined formulations	ECM complexes	Potential synergistic effect; controlled enzymatic activity

### 2.3 Advantages over conventional ESD

By replacing mechanical dissection with controlled enzymatic tissue loosening, drug-targeted endoscopic submucosal dissection (DT-ESD) has the potential to address several limitations of conventional ESD. Theoretically, this approach may reduce thermal injury, simplify technical maneuvers, and shorten procedural time by minimizing the reliance on electrosurgical tools. Moreover, if enzyme action can be precisely localized to the submucosa, the risks of perforation and bleeding could be significantly reduced.

A comparative analysis of key procedural, economic, and practical parameters between conventional ESD, Water-Jet Assisted ESD, and the proposed DT-ESD is provided in Table 2. While conventional ESD remains the gold standard, its limitations in technical complexity and procedural duration are well-known. WJ-ESD effectively addresses the issue of thermal injury and excels in specific scenarios like fibrosis, but it remains an adjunctive mechanical technology that does not fundamentally simplify the dissection process and requires significant capital investment.

**Table 2:** Comparison of conventional, water-jet, and drug-targeted ESD techniques.

Parameter	Conventional ESD	Water-jet assisted ESD	Proposed DT-ESD
Tissue separation mechanism	Electrosurgical dissection	Mechanical hydrodissection with high-pressure saline	Controlled enzymatic degradation of ECM
Thermal injury	Present (inherent to method)	Minimized (non-thermal dissection)	Minimized (non-thermal process)
Procedure duration	Long (40–120 min)	Moderate to long (reduces time for initial access)	Potentially shortened (chemical facilitation)
Technical difficulty	High (steep learning curve)	High (requires mastery of jet control)	Potentially reduced (simplifies dissection step)
Capital and procedural cost	Low capital cost (standard generator)	High capital cost (specialized pump system); moderate per-procedure cost	Low capital cost; potentially high per-procedure cost (biologic agent)
Key advantages	• Gold standard • High *en bloc* resection rates • Excellent pathological preservation	• Excellent for fibrotic lesions and initial access • Clear visual field • Avoids thermal injury	• Potential paradigm shift • May lower technical barrier • Continuous chemical “dissection”
Key limitations/downsides	• Perforation & bleeding risks • Long procedure time • Steep learning curve	• Does not replace electrosurgery for vessel sealing • Risk of uncontrollable deep dissection	• Regulatory complexity (combination product) • Potential compromise of pathological staging • Novel safety risks (diffusion, immunogenicity)
Pathological evaluation	Excellent	Excellent	Potentially compromised (requires hybrid strategy)
Training prerequisites	Extensive training required (>30–50 cases)	Additional training for jet dynamics	New training on enzymatic safety protocols; potentially simpler core dissection

In this context, DT-ESD aims to introduce a paradigm shift from mechanical to biochemical dissection. Its theoretical principal advantages include a potential reduction in technical difficulty and procedure time. However, this innovative approach introduces a distinct set of challenges, primarily its status as a combination product with a complex regulatory pathway, the potential impact on pathological specimen evaluation, and novel safety considerations regarding enzyme diffusion and immunogenicity. This comparison underscores that DT-ESD is not a direct replacement but a potential alternative with a unique risk–benefit profile, likely to find its initial niche in non-fibrotic lesions where simplifying the dissection process is the primary goal.

### 2.4 Potential risks and mitigation strategies

The theoretical implementation of DT-ESD presents several foreseeable challenges, which necessitate proactive mitigation strategies. The primary identified risks and corresponding countermeasures are outlined below.

- Excessive enzyme diffusion: Mitigated by a multi-pronged strategy: (1) physical confinement via the pre-lift and boundary incision; (2) the use of engineered viscous or thermosensitive hydrogel vehicles to restrict passive spread; and (3) the availability of a rapid-deactivation mechanism. As a critical fail-safe, a “safety wash” protocol is established, wherein the field is irrigated with a specific enzyme inhibitor (e.g., EDTA for metalloproteases) immediately post-dissection. This instantly neutralizes any residual unbound enzyme activity, providing active temporal control and terminating the digestion process on demand.
- Muscle layer injury: To prevent inadvertent injury to the muscularis propria, precise control over injection depth is paramount. This can be further safeguarded by formulating the enzymatic agent in a viscous or rheologically engineered vehicle that resists vertical seepage, thereby enhancing layer specificity.
- Delayed healing: The potential for enzymatic dissection to impair wound healing at the resection site requires thorough evaluation. Should this risk materialize, a potential management strategy involves the co-delivery of topical anti-inflammatory or pro-healing agents alongside the enzymatic formulation to promote normal tissue repair.
- Allergic or immunologic reactions: The risk of immunogenic responses can be minimized by prioritizing the use of highly purified recombinant human enzymes or engineered analogs with low immunogenicity, thereby reducing the potential for host recognition and reaction.
- Systemic exposure: The potential for systemic absorption of enzymes, though limited by the localized nature of submucosal injection, is acknowledged. This risk is primarily mitigated through the multi-pronged diffusion-control strategy outlined above, which ensures enzyme retention within the target compartment. Furthermore, the use of high-molecular-weight vehicle complexes is designed to impede vascular uptake. The rapid inactivation protocol serves as a final safeguard to neutralize any residual enzyme before it can access the systemic circulation.

To cohesively address the most critical risk of enzyme diffusion, a defense-in-depth strategy is proposed to ensure precise spatiotemporal control. This integrated framework comprises three synergistic layers of containment:

- Primary physical confinement: The foundational barrier is established by standard ESD techniques. The submucosal fluid cushion (pre-lift) and the circumferential mucosal incision collectively create a defined, relatively closed anatomical compartment. This physically delimits the operative field and serves as the first line of defense against widespread enzyme dissemination.
- Formulation-based spatial localization: The enzymatic agents are engineered into advanced delivery vehicles, such as thermosensitive or shear-thinning hydrogels. These biomaterial-based systems are designed to exhibit low viscosity during injection for ease of delivery but rapidly undergo a phase transition to a gel state *in situ* at body temperature. This transformation drastically reduces passive diffusion, effectively entrapping the enzymes within the targeted submucosal space.
- Active temporal control and a safety switch: As a critical fail-safe mechanism, a protocol for rapid enzymatic deactivation is established. Immediately following the dissection phase, the field is irrigated with a specific inhibitor (e.g., EDTA for metalloproteases). This deliberate protocol instantly neutralizes any residual, unbound enzymatic activity, providing active temporal control to terminate the digestion process on demand. This final layer prevents potential delayed complications arising from undetected micro-diffusion.

### 2.5 Clinical scenarios and applications

- Colorectal or gastric ESD in fibrotic lesions: Where mechanical dissection is challenging.
- Training scenarios: Simplifies initial dissection for trainees.
- Hybrid ESD/EMR approaches: May bridge the gap between the two.

## 3 Principles for selecting submucosa-targeted agents

The success of DT-ESD hinges on the selective disruption of the submucosal layer while minimizing collateral injury to the overlying mucosa and underlying muscularis propria. This section outlines theoretical and practical principles for guiding the selection and development of submucosa-targeted pharmacological agents.

### 3.1 Layer-specific targeting based on tissue composition

The biological rationale for drug-targeted submucosal dissection is fundamentally rooted in the unique histoarchitectural properties of the gastrointestinal wall. The submucosa constitutes a distinct, relatively acellular layer dominated by a dense network of extracellular matrix (ECM) components, most notably type I and III collagen fibrils and hyaluronic acid. This specific biochemical composition, coupled with its loose, scaffold-like structure, renders the submucosa exceptionally susceptible to targeted enzymatic degradation.

This susceptibility stands in stark contrast to the properties of the adjacent layers. The overlying mucosa is primarily a cellular epithelium with a basement membrane, while the underlying muscularis propria is a robust, cohesive layer composed chiefly of smooth muscle bundles, wherein collagen acts as a secondary, supportive stroma. Consequently, the muscularis propria possesses inherent resistance to enzymatic dissection, requiring orders-of-magnitude greater exposure time or enzyme concentration to suffer structural compromise comparable to that of the submucosa. Furthermore, the muscularis mucosae—a thin band of smooth muscle at the base of the mucosa—serves as a critical anatomical and mechanical boundary, further enhancing the potential for layer-selective intervention.

Guided by this principle of differential vulnerability, the selection of enzymatic agents is directed toward the core structural elements of the submucosal ECM:

- Collagenase (e.g., clostridial or recombinant): Targets and cleaves the native triple-helical structure of type I and III collagen, thereby directly dismantling the primary tensile framework of the submucosa.
- Hyaluronidase: Acts upon hyaluronic acid, a key glycosaminoglycan in the ground substance. Its digestion disrupts the hydrogel-like environment, significantly increasing tissue permeability and facilitating the dispersion of fluid and other agents, which collectively loosens the ECM architecture.
- Elastase: Serves as an adjunctive enzyme for specialized cases, as it degrades elastin fibers. This activity may be particularly useful for managing fibrotic submucosal tissue, where elastin content can be elevated.

### 3.2 Pharmacodynamic and kinetic criteria

Ideal agents should exhibit:

- Localized enzymatic activity with limited lateral or vertical diffusion.
- Short half-life or controlled inactivation after achieving the desired effect.
- Enzymatic activity that can be rapidly neutralized using specific inhibitors (e.g., EDTA for metalloproteinases) or by thermal denaturation at mild temperatures (e.g., 45–50 °C), offering a potential safety switch in case of unintended spread.
- Enzymatic activity at physiological pH (6.5–7.4), ensuring compatibility with gastric and intestinal tissue environments.

### 3.3 Safety and biocompatibility requirements

Selected agents should meet the following conditions:

- Low immunogenicity, preferably using recombinant human enzymes or modified bacterial enzymes.
- Minimal cytotoxicity to epithelial or muscular cells.
- Potential reversibility, via enzyme inhibitors or flushing with saline in case of inadvertent diffusion.
- Preservation of submucosal vasculature and neural plexuses to minimize postoperative bleeding, pain, or motility disorders.

### 3.4 Formulation and delivery optimization

To maximize submucosal selectivity:

- Enzymes should be formulated in moderately viscous vehicles to restrict spread and ensure retention within the submucosal layer.
- Co-formulation with contrast or dye tracers may assist in real-time visualization of agent dispersion.
- Compatibility with endoscopic injection systems and submucosal hydrodissection is essential.

### 3.5 Preclinical evaluation criteria

To ensure safety, efficacy, and tissue specificity, the preclinical development of submucosa-targeted enzymatic agents must follow a structured and multidimensional evaluation framework. This includes not only assessing the biochemical properties of the agent, but also its spatial distribution, dissection-facilitating capacity, and histopathological effects on gastrointestinal tissue. These criteria are essential for screening candidate agents and optimizing their delivery systems before *in vivo* application. The key evaluation parameters and corresponding assessment methods are summarized in Table 3.

**Table 3:** Preclinical evaluation criteria for submucosa-targeted agents.

Criterion	Description	Evaluation method
Tissue selectivity	Selective degradation of the acellular submucosal ECM with minimal architectural impact on the muscularis propria and mucosa	- Histological scoring of *ex vivo* tissue (H&E, Masson’s trichrome). - Quantification of layer-specific architectural preservation.
Facilitation of dissection	Limited vertical and horizontal spread of enzymatic activity from the injection site	- Measurement of dissection force using a force probe in *ex vivo* models. - Timing of blunt dissection in porcine ESD models.
Diffusion control	Limited vertical and horizontal spread of enzymatic activity from the injection site	- Tracer-assisted imaging (e.g., fluorescence) to quantify enzyme distribution. - Histological measurement of the digestion boundary.
Enzyme dosage and kinetics	Determination of the optimal concentration, volume, and time-action profile for effective and safe dissection	- In vitro dose–response assays against purified ECM substrates. - Time-course studies in *ex vivo* tissue to establish the minimum effective concentration and the onset/offset of action.
Inflammatory response	Low acute and chronic inflammatory reaction at the resection site	- Histopathology for immune cell infiltration. - Cytokine profiling (e.g., IL-1β, TNF-α) in tissue supernatants.
Onset/duration window	The time window for achieving effective dissection, balancing efficacy and safety	- Sequential biopsies during time-course studies to correlate enzymatic exposure time with the degree of tissue loosening and off-target effects.

### 3.6 Candidate enzymes for DT-ESD development

While several enzymes may be theoretically suitable for submucosal matrix digestion (see Table 1), not all of them are appropriate for clinical translation. Factors such as immunogenicity, availability of pharmaceutical-grade formulations, regulatory history, and safety profiles must also be considered. Table 4 summarizes a refined list of candidate agents with practical potential for DT-ESD development, along with their mechanisms, relative suitability, and relevant clinical notes.

**Table 4:** Candidate enzymes for DT-ESD development.

Agent	Mechanism	Suitability	Notes
Clostridial collagenase	Degrades collagen	High	Strong matrix action
Recombinant collagenase	ECM-selective proteolysis	Medium	Lower immunogenicity
Hyaluronidase	Degrades hyaluronic acid	Medium	Enhances matrix permeability
Elastase	Degrades elastic fibers	Low–medium	Useful in fibrosis models
Protease cocktails	Broad ECM breakdown	Variable	Risk of excessive tissue damage

### 3.7 Considerations for enzyme dosage and kinetics

The translational feasibility of DT-ESD hinges on the precise determination of enzymatic dosage (concentration and volume) and kinetic parameters (time-action profile). Initial concentration ranges for candidate enzymes will be derived from a twofold strategy: (1) benchmarking against known safe and effective concentrations used in analogous clinical applications (e.g., collagenase clostridium histolyticum formulations), and (2) establishing a dose–response curve in *in vitro* assays using purified type I/III collagen and hyaluronic acid substrates. The optimal concentration must achieve sufficient submucosal digestion within a target window of 2–10 min while preserving a high therapeutic index against off-target toxicity on muscularis propria. The injection volume, while initially estimated at 2–5 mL per quadrant, is fundamentally a delivery parameter to ensure complete lesion coverage via the multi-point injection strategy; its optimization will focus on achieving uniform dispersion without inducing high interstitial pressure. Crucially, the definition of an activity threshold, the minimal enzymatic activity per unit volume of tissue required to facilitate dissection with minimal mechanical force, will be a central deliverable of the preclinical pipeline. The key evaluation parameters and corresponding assessment methods are summarized in Table 3. This kinetic profile, rather than a fixed incubation time, will ultimately guide clinical protocol development, ensuring efficacy while containing procedural duration.

### 3.8 Future directions

To achieve next-generation tissue-specific dissection, further development may focus on:

- Bioengineered enzyme mimetics with substrate-specific affinity.
- Stimuli-responsive hydrogel carriers, activated by pH or temperature.
- Nanoparticle-encapsulated proteases with controlled release kinetics.

These directions may ultimately support the creation of precision-controlled chemical dissection tools that integrate with next-generation endoscopic platforms.

## 4 Technical challenges and future drug selection strategies

While enzyme-assisted submucosal dissection presents a promising theoretical framework, the feasibility and safety of this approach critically depend on the availability of submucosa-targeted pharmacological agents. The primary technical bottleneck lies in identifying compounds that can achieve layer-specific extracellular matrix degradation within the submucosa, without damaging the overlying mucosa or the underlying muscularis propria. The key challenges are as follows:

### 4.1 Submucosal specificity

The ideal agent must selectively degrade submucosal collagen and matrix polysaccharides while sparing epithelial and muscular structures. Many natural proteases act broadly across tissue types and lack inherent layer specificity, posing risks of perforation or delayed bleeding if not tightly controlled.

### 4.2 Vertical diffusion and depth control

Because the submucosa is tightly apposed to the muscularis propria, even minimal downward diffusion may result in full-thickness injury. Effective spatial confinement of enzymatic activity must therefore be achieved through strategies including vehicle design, dosage control, or self-limiting enzymatic kinetics.

### 4.3 Balancing activity and safety

Agents must be potent enough to loosen the submucosa within minutes, yet rapidly deactivated or cleared to prevent off-target effects. This requires fine-tuning of enzyme half-life, pH sensitivity, and compatibility with physiological fluids.

### 4.4 Absence of standardized screening platforms

There are currently no established *in vitro* or *ex vivo* models for evaluating “dissection efficiency” or “layer selectivity.” To address this, we are actively developing a two-tier screening platform combining *ex vivo* porcine intestinal tissue (harvested within 4 h of slaughter and preserved in oxygenated saline) and 3D-printed gut-mimicking phantoms composed of gelatin-collagen composite layers with tunable stiffness and dye-coded tissue analogs. The *ex vivo* porcine model enables evaluation of tissue penetration, mechanical dissection resistance, and mucosa–submucosa selectivity. In contrast, the phantom system allows for reproducible testing of enzyme diffusion kinetics under simulated endoscopic conditions. These platforms will provide quantifiable parameters for enzyme efficacy, spatial specificity, and dissection facilitation, supporting preclinical agent screening and optimization.

### 4.5 Proposed stepwise screening pipeline

To address these challenges systematically, we propose a structured five-phase drug discovery framework tailored explicitly for submucosa-targeted agents. This pipeline integrates molecular screening, preclinical validation, and delivery optimization to ensure both efficacy and safety. The detailed phases, objectives, and methodologies are outlined in Table 5. Each phase is designed to progressively evaluate biochemical, spatial, and functional properties of candidate agents, with increasing model complexity and translational relevance.

- Phase 1 (agent identification) involves bioinformatic screening and database mining to shortlist enzymes with ECM-degrading potential. This phase typically lasts 1–2 months.
- Phase 2 (substrate testing) includes *in vitro* enzymatic assays against purified type I/III collagen and hyaluronic acid substrates to assess specificity and kinetics. Estimated duration: 2–3 weeks per enzyme.
- Phase 3 (specificity validation) utilizes *ex vivo* GI tissues to visualize and quantify layer-selective digestion, using immunostaining and confocal microscopy. Duration: 4–6 weeks.
- Phase 4 (delivery optimization) tests various vehicles (e.g., viscous gels, nanoparticles) for diffusion control using fluorophore-labeled enzymes and phantom models.
- Phase 5 (*in vivo* practice) applies the best-performing formulations in live porcine ESD procedures under anesthesia, followed by histopathology and safety monitoring (8–12 weeks).

**Table 5:** Five-phase screening pipeline for submucosa-targeted agents.

Phase	Objective	Approach
1. Agent identification	Select ECM-degrading enzymes	Database mining
2. Substrate testing	Assess collagen I/III specificity	ECM-degrading *in vitro*
3. Specificity validation	Test on *ex vivo* GI tissue	Fluorescence staining, penetration assays
4. Delivery optimization	Limit diffusion	Vehicle rheology, imaging-guided injections
5. Practice *in vivo*	Assess feasibility and safety	ESD and pathological analysis

This progressive and multidimensional pipeline ensures candidate agents are not only biochemically active but also spatially selective, delivery-compatible, and safe for translational use.

### 4.6 Validation of specificity and control strategies

- The theoretical multi-layered control strategy, while comprehensive, requires rigorous empirical validation to ensure its practical efficacy. Each tier presents potential failure modes that must be scientifically addressed.
- Physical confinement: The efficacy of the fluid cushion and boundary incision in creating a closed compartment may be compromised in fibrotic tissue or due to technical variability. Validation Approach: The integrity of physical confinement will be quantified using *ex vivo* models by co-injecting fluorescent tracers with the enzyme formulation. The spatial distribution and leakage of the tracer will be imaged and measured to assess the success of compartmentalization.
- Formulation-based localization: The performance of engineered hydrogels *in vivo* must be confirmed. Variables such as tissue compliance and temperature may alter their retention properties. Validation Approach: The diffusion kinetics of enzyme-loaded vehicles will be characterized using artificial tissue phantoms with tunable stiffness and in *ex vivo* organs. Key parameters like the diffusion coefficient and the effective retention time within the submucosa will be determined.
- Active temporal control: A critical question is the ability of a neutralizing agent to control the enzymatic process once initiated. While such a wash is highly effective against unbound enzyme in the interstitial space, the primary driver of distal diffusion, its capacity to instantaneously halt the activity of enzyme molecules already bound and actively cleaving ECM substrates may be limited.
- Validation approach: The efficacy of the protocol will be tested in a time-course manner. *ex vivo* tissues will be subjected to enzymatic digestion for varying durations, followed by the application of the inhibitor. The subsequent cessation of tissue weakening will be measured mechanically (e.g., with a force probe), and the histological extent of digestion will be analyzed to define the “point of no return” and the window of effective intervention.

## 5 Pathological evaluation challenges and mitigation strategies

A paramount consideration in the development of DT-ESD is its potential impact on the histopathological assessment of the resected specimen, which is the cornerstone of oncological management. The enzymatic dissolution of the submucosal extracellular matrix (ECM) poses a unique challenge, as it may compromise the architectural integrity required for accurate staging. This section delineates the specific risks and outlines a comprehensive mitigation framework.

### 5.1 Specific risks to pathological assessment

Evaluation of deep margins and depth categorization: The definitive pathological assessment of the deep (vertical) resection margin is critical. Enzymatic degradation of the submucosal interface can blur the demarcation between the lesion and the underlying muscularis propria, making it difficult to determine whether the deep margin is free of tumor and to accurately measure the depth of submucosal invasion (e.g., distinguishing T1a from T1b lesions in early cancer). This distinction directly influences subsequent management decisions, including the need for additional surgery.

Assessment of lymphovascular invasion (LVI): The identification of tumor cells within endothelial-lined lymphatic or vascular channels is a key prognostic factor. Excessive digestion could potentially damage the architecture of these delicate channels, leading to either false-positive interpretations (due to artifactually displaced cells) or, more worryingly, false-negative results if the channels are destroyed and the evidence of LVI is obliterated.

### 5.2 A multi-tiered mitigation framework

To safeguard pathological evaluability, we propose a proactive, multi-pronged strategy:

Strict indication and pre-procedural staging: The primary mitigation is rigorous patient selection. In its initial application, DT-ESD should be reserved for lesions with a very high pre-procedural confidence of mucosal confinement (e.g., high-grade dysplasia, large sessile serrated lesions without pit pattern suggesting invasion). The use of high-resolution endoscopy, enhanced imaging, and endoscopic ultrasound (EUS) is mandatory to exclude lesions with suspected submucosal invasion, for which DT-ESD would be relatively contraindicated.

The hybrid ESD strategy: To proactively address pathological concerns, a formalized Hybrid ESD technique is recommended for cases with any diagnostic uncertainty. This strategy strategically segregates the specimen into two functional zones:

- The “Diagnostic Zone” (peripheral and deep margins): Conventional electrosurgical dissection is meticulously used to harvest the peripheral and deep margins of the specimen. This ensures that the histopathological integrity of the critical resection boundaries is entirely preserved, allowing for definitive assessment of en bloc resection completeness (R0 status) and the deepest point of invasion at the margins.
- The “Facilitation Zone” (central submucosa): Enzymatic digestion is selectively applied only to the central portion of the submucosal bed. While this may compromise the architectural evaluation of the submucosa directly beneath the lesion center, it serves the primary purpose of enabling efficient tissue loosening and resection.

This approach acknowledges that the oncological decision-making primarily hinges on margin status and the detection (rather than the ultraprecise measurement) of deep submucosal invasion. If invasion is detected in the central zone, even if its exact depth is obscured, it signals the need for further surgical management. Thus, the hybrid model strategically balances procedural facilitation with the preservation of diagnostically indispensable information.

### 5.3 Future development of pathology-friendly formulations

A long-term solution lies in the bioengineering of next-generation enzymatic agents. The ideal formulation would possess high specificity for acellular ECM components (e.g., collagen fibrils) while sparing cellular structures and the integrity of vascular and lymphatic channels. Achieving this “differential digestion” would minimize the impact on the histological features essential for accurate staging.

## 6 Discussion

For the specific challenge of fibrotic lesions, where fluid cushion creation may be suboptimal, we fully acknowledge that pure enzymatic dissection might be unreliable. Ideally, the enzyme formulation itself can be optimized. The use of combination cocktails (e.g., collagenase + hyaluronidase) and penetration-enhancing carriers is anticipated to improve tissue permeation and distribution, overcoming the diffusion barriers presented by dense fibrosis. In turn, the procedure would seamlessly transition to the previously described “Hybrid ESD” strategy. The enzymatic approach would be applied only to areas where a cushion can be formed, while fibrotic bands and critical margins would be addressed with conventional electromechanical dissection.

One of the most critical considerations for the proposed DT-ESD approach is its potential impact on pathological staging integrity. The procedure’s diagnostic utility hinges on the ability to perform an accurate histopathological evaluation post-resection. In early gastrointestinal cancers, where therapeutic decisions are guided by precise substaging, such as differentiating between T1a (mucosal) and T1b (submucosal) invasion, assessing lymphovascular involvement, and determining resection margin status, the architectural preservation of the *en bloc* specimen is paramount.

The enzymatic digestion central to DT-ESD inherently poses a challenge to this requirement. The dissolution of the submucosal extracellular matrix (ECM) can obscure subtle histological landmarks, potentially compromising the evaluation of deep invasion fronts and critical margins. To proactively address this fundamental concern, a multi-tiered, defensive strategy is essential for the responsible clinical translation of DT-ESD.

First, stringent patient and lesion selection serves as the primary safeguard. DT-ESD should be preferentially applied to lesions with a high pre-procedural confidence of confinement to the mucosal layer. Ideal candidates include low-grade adenomas, high-grade dysplasia, or large sessile serrated lesions without endoscopic features suggestive of submucosal invasion. For any lesion raising suspicion, pre-procedural staging with endoscopic ultrasound (EUS) or advanced optical imaging is mandatory to exclude deep invasion, effectively contraindicating pure enzymatic dissection in such cases.

Second, for cases where any diagnostic uncertainty persists, or to universally maximize pathological evaluability, a formalized “Hybrid ESD” technique is proposed. This approach strategically integrates both conventional and novel methods: conventional electrosurgical dissection is meticulously used to define and secure the peripheral and deep margins of the specimen. This ensures that the diagnostically most critical areas for assessing *en bloc* resection completeness and deep vertical invasion remain entirely unaffected by enzymatic activity. Enzymatic digestion is then deployed selectively only within the central submucosal space, solely to facilitate the final lifting and separation of the lesion body. This hybrid model thus strategically balances the procedural ease offered by enzymatic digestion with the uncompromised pathological assessment required for oncological management.

Finally, looking forward, the development of next-generation, “pathology-friendly” enzymatic formulations represent a promising direction. Future research should focus on engineering enzyme cocktails or mimetics with enhanced selectivity for acellular ECM components (e.g., specific collagen types) while sparing cellular structures and vascular or lymphatic walls. Achieving such biochemical precision would further minimize the impact on histopathology, ultimately strengthening the diagnostic robustness of the DT-ESD paradigm.

## 7 Future perspectives

The success of DT-ESD depends critically on the identification of pharmacological agents that are both effective and selective for the submucosal layer. Agent selection is the cornerstone of this approach, requiring enzymes with high matrix specificity and minimal off-target effects. Preclinical studies using *ex vivo* and porcine models will be essential to evaluate dissection efficiency, safety, and tissue selectivity. In parallel, optimizing dosage, exposure time, and diffusion control will help reduce complications. Collaboration with bioengineers will facilitate the development of targeted delivery systems and self-limiting formulations. Comprehensive toxicological profiling is also necessary to ensure both acute and long-term safety. The development of DT-ESD is inherently interdisciplinary, requiring a close collaboration between gastroenterology, enzymology, and biomaterial engineering.

The transition of DT-ESD from a theoretical concept to a clinically viable technique hinges on a structured and rigorous preclinical validation process. The multi-phase screening pipeline outlined in Table 5 is designed to systematically address the core challenges of agent specificity, safety, and delivery. This structured, multi-phase pipeline is proposed as a definitive translational roadmap to systematically de-risk the development of DT-ESD. Our immediate future work will focus on executing the initial phases of this pipeline (Agent Identification to Specificity Validation) to identify the most promising candidate formulations for subsequent *in vivo* testing.

## 8 Regulatory pathway and clinical translation considerations

The translational pathway for DT-ESD, classified as a drug-led combination product, necessitates a rigorous and integrated regulatory strategy with regulatory bodies such as the U.S. FDA or EMA. The journey from preclinical validation to clinical application will be structured around a phased first-in-human (FIH) study protocol, designed to maximize safety and efficiently establish proof-of-concept.

- FIH clinical trial design: Clinical development will likely commence with a Phase 1b/2a trial in patients with well-defined, accessible superficial gastrointestinal neoplasms amenable to endoscopic resection. A preceding Phase 1a study in healthy volunteers may be considered if supported by toxicology data, to assess the systemic pharmacokinetics and safety of the enzyme formulation alone.
- Dose-escalation methodology: The core of the initial clinical trials will be a structured dose-escalation phase. We propose employing a modified 3 + 3 design or a model-based adaptive design [e.g., Bayesian Logistic Regression Model (BLRM)]. The primary objectives will be to determine the Minimum Effective Dissection Dose (MEDD)—defined as the lowest dose that achieves satisfactory submucosal lifting and separation with minimal mechanical assistance—and the Maximum Tolerated Dose (MTD), identified by the occurrence of dose-limiting toxicities (e.g., clinically significant perforation or bleeding attributable to the enzyme). Escalation will be guided by a pre-specified Safety Review Committee.
- Comprehensive safety surveillance system: A multi-layered safety surveillance system will be implemented, including:

- Intraprocedural safety: Continuous monitoring for immediate adverse events (AEs) such as unintended muscle layer injury or bleeding.
- Short-term follow-up: Standardized endoscopic assessment of the ulcer bed at 24–48 h post-procedure to evaluate for delayed perforation or bleeding.
- Long-term follow-up: Planned endoscopic re-evaluation at 4 and 12 weeks to monitor wound healing, assess for stricture formation, and confirm the absence of local recurrence.
- Data collection: All AEs will be recorded and graded according to established scales (e.g., CTCAE), with particular attention to characterizing the local tissue response to the enzymatic agent.

Successfully navigating this pathway will require extensive preclinical data packages for both the biologic and its delivery system, but this structured approach provides a clear framework for de-risking the clinical translation of DT-ESD.

## 9 Conclusion

Drug-targeted endoscopic submucosal dissection (DT-ESD) presents a novel theoretical strategy for chemically guided tissue separation, aiming to overcome key limitations of conventional ESD, such as technical complexity, procedural duration, and thermal injury. By selectively degrading the submucosal matrix, DT-ESD may reduce reliance on electrosurgical tools and simplify training. Clinically, it holds particular promise for fibrotic lesions and trainee-friendly procedures, potentially expanding access to advanced endoscopic therapy. While still in the conceptual stage, DT-ESD reflects a broader evolution in therapeutic endoscopy, from mechanical to biochemical precision, and may represent a future direction in “third-generation endoscopy tools.” Preclinical studies are warranted to evaluate its safety, efficacy, and translational potential.

## Data Availability

The original contributions presented in the study are included in the article/supplementary material, further inquiries can be directed to the corresponding authors.
